# Neuropeptide Y mRNA expression in the aging inferior colliculus of fischer brown norway rats

**DOI:** 10.3389/fnagi.2025.1626021

**Published:** 2025-07-23

**Authors:** Laila S. Almassri, Kristen M. Crane, Sean R. Hergenrother, Gurveer Singh, Gillian L. Barach, Melina C. Iafrate, Joshua C. Harris, Nick Tokar, Andrew P. Ohl, Jesse W. Young, Jeffrey G. Mellott

**Affiliations:** ^1^Department of Biomedical Sciences, Northeast Ohio Medical University, Rootstown, OH, United States; ^2^Department of Biomedical Sciences, University Hospitals Hearing Research Center, Northeast Ohio Medical University, Rootstown, OH, United States

**Keywords:** inferior colliculus, NPY, smFISH, NPY mRNA, GAD1 mRNA, aging

## Abstract

**Introduction:**

A major contributor to age-related hearing loss is the decline of GABAergic inhibition, particularly in the inferior colliculus (IC), which is the midbrain hub of the central auditory system. The initial loss of inhibition is thought to be a compensatory mechanism in response to decreased peripheral excitation. However, the downregulation of inhibition in the IC persists with age and leads to functional disruptions and central neural gain. Neuropeptide Y (NPY) is co-expressed by a sub-population of GABAergic IC cells whose age-related changes remain unexplored. We sought to characterize GABAergic cells in the major subdivisions of the IC that express NPY mRNA to determine whether NPY mRNA is altered in aging IC cells.

**Methods:**

We used multiplexed fluorescent *in situ* hybridization (smFISH) to label lemniscal and non-lemniscal IC cells that express NPY mRNA and/or GAD1 mRNA in four age groups of Fischer Brown Norway (FBN) rats.

**Results:**

The data demonstrate that ∼38% of GABAergic IC cells co-express NPY, the largest proportion of NPY cells is in the non-lemniscal dorsal IC (ICd), the majority of NPY cells have medium profile areas, and the expression of individual NPY mRNA is unaffected by age.

**Discussion:**

While GABAergic inhibition is reduced with age, it appears that NPY driven inhibition may remain intact. GABAergic neurons that co-express NPY may represent a marked population that persists throughout aging, suggesting that they are not the primary contributor to age-related loss of inhibition.

## 1 Introduction

Age-related hearing loss (ARHL), a commonly reported chronic health condition amongst the elderly population, impacts an individual’s ability to communicate and poses serious health risks (Goman and Lin, 2016; Haile et al., 2021; Man et al., 2021). Diminished ability to hear high frequency sounds, decreased speech understanding, and impaired sound localization are several of the hallmarks of ARHL, which encompass a combination of peripheral and central auditory deficits (Ibrahim and Llano, 2019; Li-Korotky, 2012; Ouda et al., 2015). The central neural gain hypothesis suggests that decreased excitation from the periphery leads to a compensatory downregulation of GABAergic inhibition centrally, particularly in the auditory midbrain (Ono and Oliver, 2014; Ibrahim and Llano, 2019; Knipper et al., 2022; Rumschlag et al., 2022; Ouda et al., 2015). Although advantageous at first, the continued loss of inhibition into old age leads to dysfunction in temporal coding, signal-to-noise ratios, and shaping of acoustic signals (Palombi and Caspary, 1996a,b; Walton et al., 1998, 2002; Frisina, 2001; Suta et al., 2011; Parthasarathy and Bartlett, 2011, Pollak et al., 2011; Cai et al., 2014; Koehler et al., 2023).

The age-related downregulation of GABA and its precursor, glutamic acid decarboxylase (GAD), are well established in the inferior colliculus (IC), which is the midbrain hub of the central auditory system (Wenstrup, 2005; Caspary et al., 2008; Syka, 2020; Caspary et al., 1990, 1995; Burianova et al., 2009; Gutiérrez et al., 1994; Raza et al., 1994; Ouda and Syka, 2012; Rabang et al., 2012; Milbrandt et al., 1994, 1996, 1997; Pal et al., 2019; Koehler et al., 2023). The IC is separated into three anatomical subdivisions across most species: the central (ICc), lateral cortex (IClc), and dorsal cortex (ICd) (see reviews: Oliver, 2005; Syka, 2020). Nearly all major ascending and descending auditory information is processed in the IC: ascending lemniscal information is largely processed in the ICc and IClc, descending cortical information in the ICd and IClc, as well as intrinsic and commissural information across the three subdivisions (González-Hernández et al., 1996; Oliver, 2005; Schofield, 2010; Thompson, 2005; Wenstrup, 2005; Mellott et al., 2014a,^2018^). The IC is comprised of GABAergic cells, approximately 20%–40% of the population, and glutamatergic cells, making up the remainder of the cell population (Peruzzi et al., 1997; Merchán et al., 2005; Mellott et al., 2014a; Schofield and Beebe, 2019). Functionally, GABAergic IC cells can be placed into loose categories based on extracellular markers and soma size (Ito et al., 2009; Beebe et al., 2016). Recent studies suggest that co-transmitter Neuropeptide Y (NPY) is expressed by a subpopulation of GABAergic neurons in the IC to inhibit local excitation (Silveira et al., 2020, 2023).

Neuropeptide Y is a 36-amino acid residue peptide that is abundant in the brain and is implicated in many physiological functions, including circadian rhythms, neuronal excitability, neuroplasticity, and memory (Gøtzsche and Woldbye, 2016). NPY is primarily expressed by GABAergic neurons, but it has also been observed in astrocytes and in circulation (Edvinsson et al., 1983; Ramamoorthy and Whim, 2008; Silveira et al., 2020). Further characterization has demonstrated that ICc NPY neurons have a stellate morphology and their dendritic arbors reach across isofrequency laminae (Drotos and Roberts, 2024; Goyer et al., 2019; Silveira et al., 2020). NPY neurons project to the contralateral IC and to the medial geniculate body (MG) of the auditory thalamus (Nakagawa et al., 1995; Silveira et al., 2020). NPY neurons represent approximately one third of the GABAergic cells in the mouse IC and the NPY Y1 receptor has been shown to be expressed by 80% of glutamatergic cells in the IC (Silveira et al., 2023). It remains to be determined whether NPY is expressed by small, medium, or large GABAergic cells. This is of interest because GABAergic IC cells have been shown to have distinct functional roles based on cell size and neurochemical profile (Beebe et al., 2016; Drotos and Roberts, 2024). Additionally, GABA and its mRNA precursor, GAD1, are differentially downregulated with age, but the age-related changes in NPY remain to be known (Wenstrup, 2005; Caspary et al., 2008; Syka, 2020; Caspary et al., 1990; Burianova et al., 2009; Ouda and Syka, 2012; Rabang et al., 2012; Koehler et al., 2023).

In this study we had two goals: (1) characterize cells that express NPY mRNA in a model commonly used in aging studies, and (2) to determine if NPY mRNA is altered during aging. We used fluorescent small molecule *in situ* hybridization (smFISH) and fluorescent Nissl staining to investigate age-related changes in the density of NPY cells, their size, and mRNA counts across the aging IC of the Fischer Brown Norway (FBN) rat of four age groups (2–3, 19, 24, and 28 months). Based on previous studies showing downregulation of inhibition in the aging IC, we hypothesized that NPY mRNA would decline with age. We investigated labeling for NPY mRNA, GAD1 mRNA, Neurotrace, and DAPI. Multiplexing for NPY and GAD1 allowed us to determine the distribution and percentage of GABAergic IC cells that co-express NPY. We found that (1) all subdivisions at each age group had many cells that express NPY, (2) the largest proportion of NPY expressing cells were found in the ICd, (3) a little over a third of GAD1 cells co-expressed NPY, (4) most NPY cell profiles were medium sized in area, which persisted throughout age, and (5) the density of mRNA puncta was commonly greater in the ICd across most age groups.

## 2 Materials and methods

### 2.1 Animals

All procedures were conducted in accordance with the Northeast Ohio Medical University Institutional Animal Care and Use Committee and NIH guidelines (IUCUC protocol number: #23-01-351). Results are described from 23 male Fischer Brown Norway (FBN) rats (National Institute of Aging; Bethesda, MD, United States; RRID:SCR_007317) across four age groups: 2–3 months “young” (eight animals); 19 months “early middle-age” (five animals); 24 months “late middle-age”(five animals); 28 months “old” (five animals; Table 1). Efforts were made to minimize the number of animals and their suffering.

**TABLE 1:** Neuropeptide Y (NPY) cell sizes across inferior colliculus (IC) subdivisions and ages.

Age (mo)	Average profile area (μ m^2^)	ICc% small NPY cells	ICc% medium NPY cells	ICc% large NPY cells	ICd% small NPY cells	ICd% medium NPY cells	ICd% large NPY cells	IClc% small NPY cells	IClc% medium NPY cells	IClc% large NPY cells
2–3	218.68	16.5	79.1	4.4	17.4	78.5	4	16	74.4	9.6
19	301.37	6	76.9	17.1	11.1	77.3	11.6	6.5	63.4	30
24	292.62	3.4	74.5	22.2	5	84.8	10.2	5.7	74.8	19.5
28	227.41	10.5	81.7	7.8	15.6	81.3	3	10	81.5	8.5

### 2.2 Perfusion and sectioning

Each animal was deeply anesthetized with isoflurane and perfused transcardially with 0.9% saline or Tyrode’s solution buffered at a pH of 7.4. The brain was removed, the frontal cortex was “blocked,” placed in a plastic cube mold, covered in optimal cutting temperature compound (OCT), flash frozen in liquid nitrogen, and stored at −80°C. The brain was then removed from the −80°C storage, the plastic mold was removed, and the cube of OCT/brain was placed in a cryostat (Leica) for 30–60 min to warm up to −20°C for sectioning. The brain was cut into 12 μm thick transverse sections and collected on Superfrost Plus slides (Fisher Scientific) and placed back into −80°C storage until reacted for single molecule fluorescent *in situ* hybridization (smFISH).

### 2.3 Tissue processing for smFISH

Mid-rostrocaudal inferior colliculus (IC) sections (between interaural levels 0.24–0.48 mm; Paxinos and Watson, 1998) were chosen in which the three major IC subdivisions [central IC (ICc), lateral cortex of the IC (IClc) and dorsal cortex of the IC (ICd)] were present. IC sections were processed for Neuropeptide Y [NPY; targeted bases 2-564, Cat. No. 1211911-C1, Advanced Cell Diagnostics (ACD), Unite States] and glutamate decarboxylase 1 (GAD1; targeted bases 62-3113, Cat. No. 316401-C2, ACD, Unite States) and a blank negative control (Cat. No. 320891, ACD, Unite States) using the Fluorescent Multiplex Reagent v2 Kit according to the manufacturer’s instructions (Cat. No. 323137, ACD, Unite States) as in Salehi et al. (2018), Mellott et al. (2022), Koehler et al. (2023). Briefly, slides with IC sections were immersed in a bath of chilled (4°C) 4% paraformaldehyde for 1 h. Tissue was then dehydrated at room temperature, stepwise from 50% to 100% with ethanol. Tissue was airdried and then a hydrophobic barrier was applied around each section. Next, RNAscope Hydrogen Peroxide was applied for 10 min at room temperature and then removed, followed by RNAscope Protease IV applied for 30 min at room temperature and then removed. The NPY and GAD1 probes were combined and added to each section and incubated at 40°C for 2 h. Slides were then submerged in RNAscope Wash Buffer and gently agitated for 2 min. Repeated washes occurred with fresh wash buffer. Development of the NPY and GAD1 mRNA signal occurred through a series of amplification steps at 40°C: Amp 1, 30 min; Amp 2, 30 min; Amp 3, 15 min. All steps at 40°C were conducted within a controlled humidity environment (HybEZ oven and humidity paper, ACD, United States) such that sections never dried out. RNAscope Horse Radish Peroxidase (HRP) was applied for 15 min at 40°C. Fluorescent opal reagent packs were then applied to each section to label NPY and GAD1 mRNA (NPY; Opal 650, Cat. No. FP1497001KT, Akoya Biosciences, United States; GAD1: Opal 780 reagent pack, Cat. No. FP1501001KT, Akoya Biosciences, United States). Sections were then washed and counter stained for DAPI and a green (500/525) Nissl stain, NeuroTrace (NT, Molecular probes, diluted 1:100), to label nuclei and cell bodies, respectively.

### 2.4 Data analysis and puncta detection

Two quadruple-labeled transverse IC sections were used for quantification per case. Each section was outlined using a Neurolucida reconstruction system (MBF Bioscience, Williston, VT, United States) attached to a Zeiss AxioImager M2 fluorescence microscope (Carl Zeiss) and Hamamatsu Orca Flash 4.0 camera (Hamamatsu). Differential patterns of neural packing density and somatic size, determined by Nissl, and the rat anatomical atlas of the brain were used to draw borders between the central nucleus (ICc) and the lateral (IClc) and dorsal (ICd) cortices of the IC (Faye-Lund and Osen, 1985; Loftus et al., 2008, Mafi et al., 2022). We initially subdivided (with Neurolucida) the ventromedial-dorsolateral axis of the ICc into three equal lengths to create anatomical regions representing the distribution of high, middle, and low frequencies. However, analysis showed no significance across these three regions regarding age-related changes in NPY mRNA puncta counts. Consistent with previous studies of NPY in the IC, we found NPY+ cell bodies in all three subdivisions of the IC (Nakagawa et al., 1995; Silveira et al., 2020, 2023).

One IC section per case was used for plotting NPY+/GAD1+ (12,546 cells were quantified), GAD1+ only (20,712 cells were quantified), and NPY+ only (979 cells were quantified). Each combination was plotted with a unique marker. Every cell that expressed NPY and/or GAD1 mRNA was manually plotted with a 40x objective aligned to a Neurolucida reconstruction system (MBF Bioscience, Williston, VT, United States) attached to a Zeiss AxioImager M2. Plots of markers and IC subdivisions were then analyzed through NeuroExplorer (MBF Bioscience, Williston, VT, United States).

**FIGURE 1:**
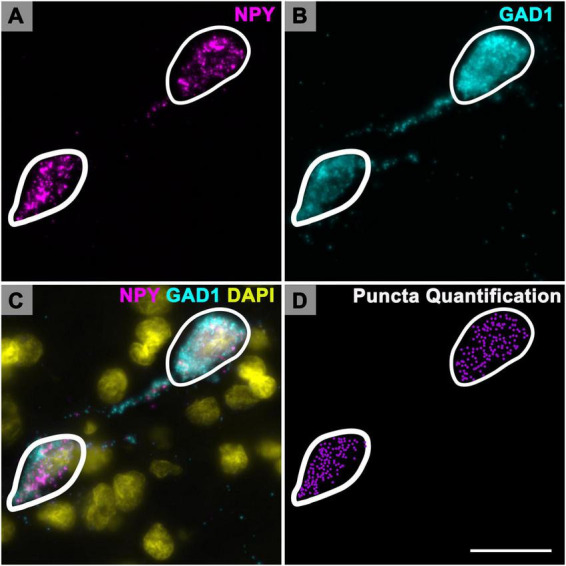
Quantification of Neuropeptide Y (NPY) messenger ribonucleic acid (mRNA) puncta in inferior colliculus (IC) cells. High magnification photomicrographs showing cells expressing NPY mRNA (magenta) and glutamate decarboxylase 1 (GAD1) mRNA (cyan) in the ICd of a middle aged (19 months) animal. At 150× magnification the individual mRNA signals are optically resolved. **(A)** Each magenta signal represents a single mRNA for NPY. **(B)** Same cells imaged to show GAD1 mRNA expression. **(C)** Merged image showing colocalization of NPY mRNA and GAD1 mRNA in each cell. DAPI (yellow) counterstain. **(D)** Plot of the same cells. White contours outline the somatic profile area of each cell (profile areas: 278.6 μm^2^, left cell; 333.9 μm^2^, right cell). Profile area was determined by counterstaining the tissue with NeuroTrace (not shown). Each purple puncta represents a single mRNA for NPY. Scale bar = 25 μm.

One IC section per case was chosen for NPY mRNA puncta quantification. Each NPY mRNA expressing cell, with a visible nucleus was contoured within Neurolucida to obtain a profile area (Figure 1). Profile area categories for this study were determined by our previous study on GAD1 mRNA (Koehler et al., 2023). Briefly, profile areas were log transformed in R to establish a quantile distribution of the lower, middle and upper third data sets that define our small (< 124 μm^2^), medium (124–411 μm^2^) and large (> 411 μm^2^) profile sizes (Koehler et al., 2023).

Neuropeptide Y mRNA puncta was manually plotted (Neurolucida) with a 150x glycerine-immersion objective (Zeiss) for each contoured cell (Figure 1D). A total of 13,452 NPY cells were contoured and had their mRNA plotted. Plots of contoured cells, mRNA puncta, and IC subdivisions were then analyzed through NeuroExplorer (MBF Bioscience, Williston, VT, United States). All photomicrographs presented here are 2–7 μm (0.2–0.5 μm steps) maximum image projections and were captured with a Zeiss AxioImager M2 fluorescence microscope and Hamamatsu Orca Flash 4.0 camera (Hamamatsu). Adobe Photoshop (Adobe Systems) was used to add scale bars, crop images, and adjust intensity levels. The results of these plots were used for a quantitative summary of the NPY mRNA across different cell sizes in different IC subdivisions, and across four age groups. Final images of the plots were refined with Adobe Illustrator (Adobe Systems, Inc., San Jose, CA, United States) for preparation of figures.

Variation in the density of mRNA, NPY+, GAD+, and GAD+/NPY+ cells was assessed using mixed-effects analyses of variance (ANOVAs), specifying age group and IC regions (nested within individual) as fixed factors, and individual animal as a random intercept in all models (see Supplementary Tables 1–4). Mixed-effects models permitted us to control for the potential pseudoreplication that would arise from analyzing repeated measurements from the same individuals (Harrison et al., 2018). To better approximate distributional normality, as assessed using quantile-quantile (QQ) plots (Sokal and Rohlf, 2011), mRNA density was square root transformed and NPY+, GAD+, and NPY/GAD+ densities were rank-transformed prior to analysis. We fit full-factorial models for each variable of interest, testing the main effect of age, the main effect of IC region, and the interaction between age and IC region. The accuracy of all mixed-effects model fits was assessed by (1) visual inspection of residual-vs-fitted value plots and (2) use of QQ plots to verify distributional normality of model residuals. Pairwise *post hoc* comparisons between age groups within IC regions or between IC regions within age groups were corrected for alpha-inflation using the False Discovery Rate method (Benjamini and Hochberg, 1995).

Significance for all tests was accepted at *p* < 0.05. All statistical analyses were conducted in the R statistical platform v. 4.4.2 “Pile of Leaves” (R Core Team, 2025), including the add-on packages emmeans (Lenth, 2024), lme4 (Bates et al., 2015), lmerTest (Kuznetsova et al., 2017), and tidyverse (Wickham et al., 2019).

## 3 Results

### 3.1 NPY mRNA is expressed in all three subdivisions of the IC in FBN rats

**FIGURE 2:**
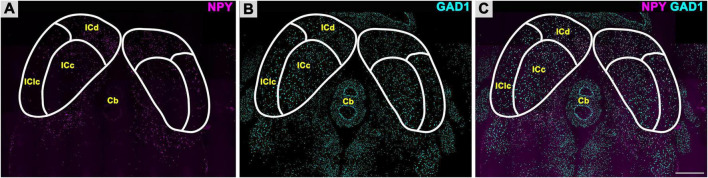
Fluorescent *in situ* hybridization for Neuropeptide Y (NPY) and glutamate decarboxylase 1 (GAD1) messenger ribonucleic acid (mRNA). NPY mRNA is expressed in all subdivisions of the inferior colliculus (IC). **(A)** Low magnification image of NPY mRNA (magenta) expression in the ICc, ICd, and IClc. **(B)** Expression of GAD1 mRNA (cyan) in the ICc, ICd, and IClc. **(C)** Merged image showing differential expression of NPY mRNA as compared to GAD1 mRNA across the IC. Scale bar = 1 mm.

To investigate the distribution of GABAergic cells that express NPY in the FBN rat IC, we performed smFISH for NPY mRNA and GAD1 mRNA. As shown in Figure 2A, NPY + mRNA puncta were observed throughout the whole IC, with a denser distribution throughout the ICd (Figure 2A). GAD1 + mRNA puncta were diffusely distributed throughout the ICd, IClc, and ICc (Figure 2B). Even from a low magnification, it was obvious that most cells expressing NPY also expressed GAD1 (Figure 2C). Overall, a total of 12,546 NPY/GAD1 cells were plotted, 20,712 GAD1 only cells were plotted, and we found that 979 cells expressed NPY without GAD1. Thus, 37.7% (12,546/33,258) of GAD1 cells also expressed NPY.

**FIGURE 3:**
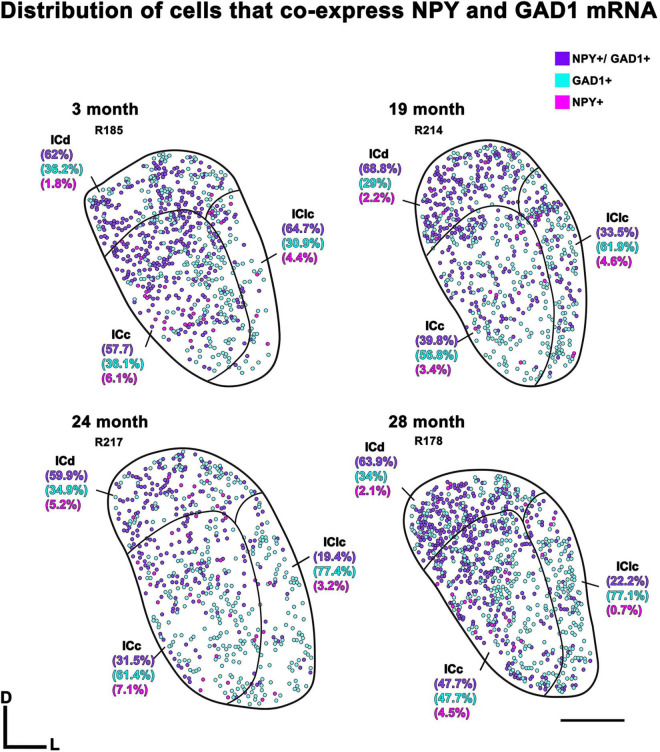
Plots showing the distribution of inferior colliculus (IC) cells that co-express Neuropeptide Y (NPY) messenger ribonucleic acid (mRNA) and glutamate decarboxylase 1 (GAD1) mRNA (purple circles), only express NPY mRNA (pink circles), or only express GAD1 mRNA (blue circles) across four age groups. Percentage of IC cells that co-express NPY mRNA and GAD1 mRNA for the individual subdivisions are shown in parentheses. Plotted sections were chosen between interaural levels 0.12–0.48 mm; (Paxinos and Watson, 1998). D, dorsal; L, lateral. Transverse sections at a mid-rostrocaudal level of the IC. Scale bar = 1 mm.

**FIGURE 4:**
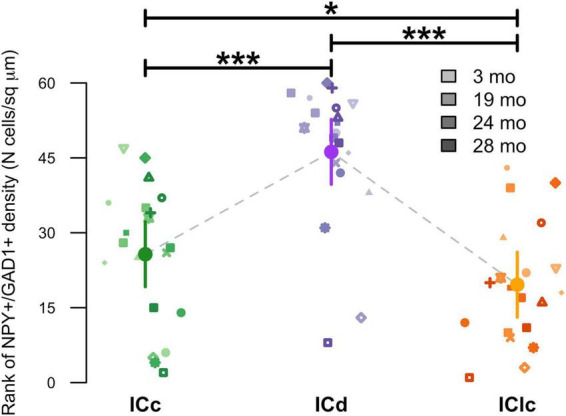
Variation in NPY+/GAD1+ density as a function of age and inferior colliculus (IC) subdivision [i.e., central (ICc), dorsal cortex (ICd), or lateral cortex (IClc)]. Raw data were rank-transformed prior to analysis due to departure from distributional normality (Wood and Games, 1990). Small markers indicate measurements of individual animals, with different symbols representing individuals and darker hues indicating older ages, as shown in the legend. Large circles with error bars represent estimated marginal means within each IC subdivision, and 95% confidence intervals around these means, respectively, as predicted by the mixed-effects regression model. Horizontal bars indicate significant differences between groups (*, *p* < 0.05; ***, *p* < 0.001).

Representative schematic plots of cells that either express both NPY and GAD1 mRNA (purple circles), only GAD1 mRNA (cyan circles), or only NPY mRNA (pink circles) in the subdivisions of the FBN rat IC at 2–3, 19, 24, and 28 months (Figure 3). Regardless of age, the ICd had the highest (∼> 60%) proportion of cells that expressed both NPY/GAD1 (Figure 3). The lowest (∼< 35%) proportion of co-expressing cells was commonly in IClc (Figure 3). The proportion of co-expressing cells in the ICc fell in the middle (Figure 3). We then examined the density of these co-expressing cells within the known area (μm^2^) of each subdivision across all animals (Figure 4). Regardless of aging, the density of cells that co-express NPY and GAD1 was significantly higher in the ICd as compared to the ICc (***p < 0.0001) and the IClc (***p < 0.0001; Figure 4). Density was higher (*p = 0.0141) in the ICc than the IClc (Figure 4).

**FIGURE 5:**
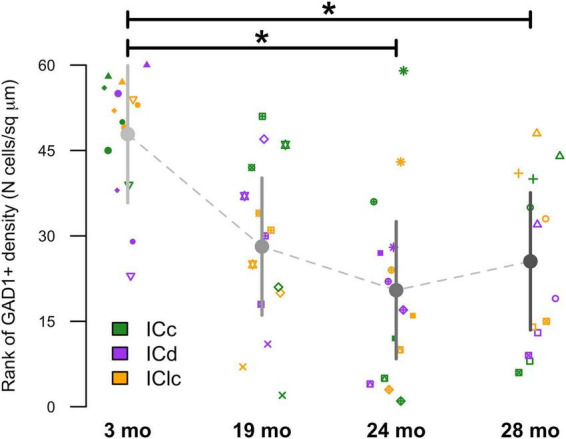
Variation in GAD1+ as a function of age and inferior colliculus (IC) subdivision [i.e., central (ICc), dorsal cortex (ICd), or lateral cortex (IClc)]. Raw data were rank-transformed prior to analysis due to departure from distributional normality (Wood and Games, 1990). Small markers indicate measurements of individual animals, with different symbols representing individuals. Large circles with error bars represent the estimated marginal means at each age, and 95% confidence intervals around these means, respectively, as predicted by the mixed-effects regression model. Horizontal bars indicate significant differences between groups (*, *p* < 0.05).

Representative plots from each age group indicated that cells only expressing GAD1 (cyan) in the ICd only account for 29%–36.2% of the examined population, while in the ICc and IClc GAD1 only cells could account for more than half of the total population (Figure 3). We then examined the density of these GAD1 only expressing cells within the known area (μm^2^) of each subdivision across all animals (Figure 5). The density of these GAD1 only cells significantly decreased from 2–3 months to 24 months (*p = 0.0216) and to 28 months (*p = 0.0403; Figure 5).

**FIGURE 6:**
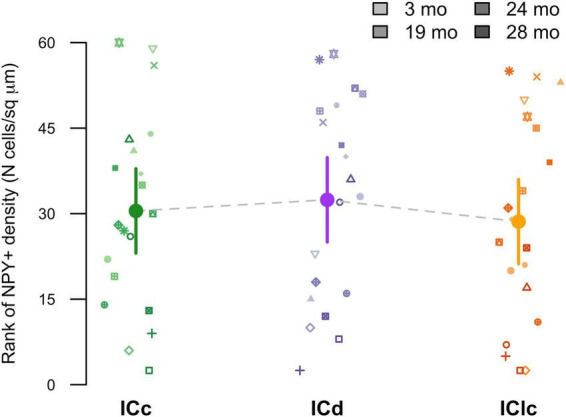
Variation in NPY+ density as a function of age and IC subdivision [i.e., central (ICc), dorsal cortex (ICd), or lateral cortex (IClc)]. Raw data were rank-transformed prior to analysis due to departure from distributional normality (Wood and Games, 1990). Small markers indicate measurements of individual animals, with different symbols representing individuals and darker sha. Large circles with error bars represent estimated marginal means at each age, and 95% confidence intervals around these means, respectively, as predicted by the mixed-effects regression model.

Representative plots from each age group indicated that cells expressing only NPY (pink) were not common in the ICd (1.8%–5.2%), the ICc (3.4%–7.1%), and the IClc (0.7%–4.6%; Figure 3). We then examined the density of these NPY only expressing cells within the known area (μm^2^) of each subdivision across all animals (Figure 6). However, there were no significant differences noted in their expression patterns (Figure 6).

### 3.2 NPY and GAD1 mRNA is co-expressed in small, medium, and large IC cells

Following our previous study on GAD1 mRNA (Koehler et al., 2023) we recognized three size groups: small cells have a profile area that is less than 124 μm^2^, medium cell profile areas ranged from 124 to 411 μm^2^, and large cells have profile areas greater than 411 μm^2^. As only GABAergic cells express NPY in the IC, we apply these profile areas to our NPY cells. In the current study, the vast majority (79%) of NPY mRNA expressing cells had a profile area between 124 and 411 μm^2^, thus we defined them as medium. The remaining 21% was split across small (10.8%) and large (10.2%) populations. When examining specific IC subdivisions, medium NPY cells were the most common size in each subdivision at each age (Table 1). The overall average profile area between ages ranged from 218.68 to 301.37 μm^2^ (Table 1). Interestingly, the average profile area at each age was that of a medium cell, although averages were larger during 19 and 24 months (Table 1).

**FIGURE 7:**
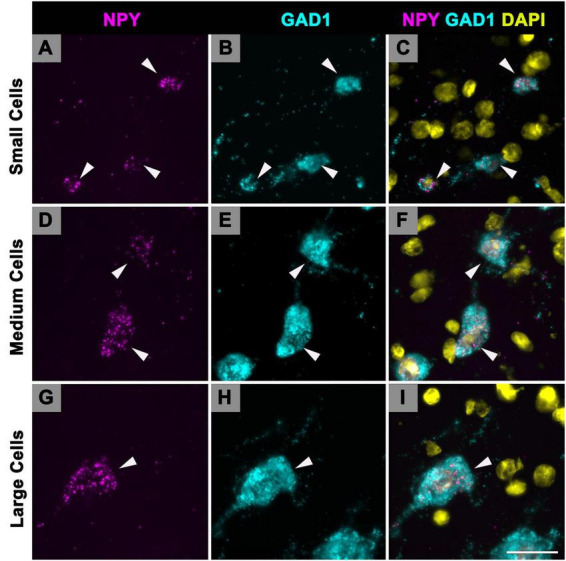
Cells that express Neuropeptide Y (NPY) messenger ribonucleic acid (mRNA) vary in soma profile area. High magnification (150×) photomicrographs showing NPY mRNA expression in small, medium, and large cells, based on soma size classification of GAD1+ cells in previous studies (small, < 124 μm^2^; medium, 124–411 μm^2^; large, > 411 μm^2^). NPY mRNA is shown in magenta, glutamate decarboxylase 1 (GAD1) mRNA is shown in cyan, and DAPI is shown in yellow. Each row represents a single cell size. **(A–C)** Photomicrographs of small cells (arrowheads) in the ICd of a young (3 months) animal (top cell, 119.6 μm^2^; middle cell, 116.5 μm^2^; bottom cell, 69.8 μm^2^). **(D–F)** Photomicrographs of medium cells (arrowheads) in the ICd of a young (3 months) animal (top cell, 233.4 μm^2^; bottom cell, 365.6 μm^2^). **(G–I)** Photomicrographs of large cells (arrowheads) in the ICc of a young (3 months) animal (center cell, 587.4 μm^2^). Scale bar = 25 μm.

Multiplexing smFISH techniques show specific examples of small, medium and large GABAergic cells that express NPY mRNA (Figure 7). Small cells (top cell, 119.6 μm^2^; middle cell, 116.5 μm^2^; bottom cell, 69.8 μm^2^) that co-express NPY and GAD1 mRNA can be readily identified in the ICd (white arrowheads; Figures 7A–C). Medium cells (top cell, 233.4 μm^2^; bottom cell, 365.6 μm^2^) that co-express NPY and GAD1 mRNA in the ICd (white arrowheads; Figures 7D–F). A large cell (587.4 μm^2^) that co-expresses NPY and GAD1 mRNA (white arrowhead; Figures 7G–I). Mixed-effect modeling did not find a significant difference of the distribution of NPY profile areas across subdivisions or ages.

### 3.3 Expression of NPY mRNA in 2–3 months old FBN rats

**FIGURE 8:**
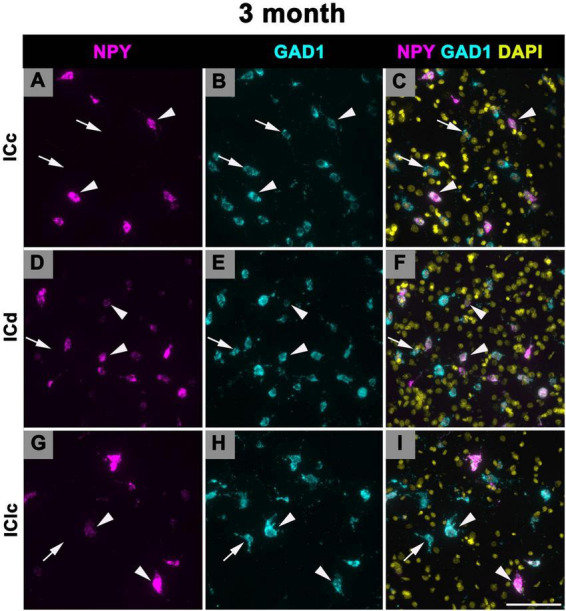
Structured illumination fluorescence images, taken at 0.2 μm steps, showing cells that co-express Neuropeptide Y (NPY) messenger ribonucleic acid (mRNA) and glutamate decarboxylase 1 (GAD1) mRNA (arrowheads) and cells that only express GAD1 mRNA (arrows) in a young (3 months) animal. NPY mRNA is shown in magenta, GAD1 mRNA is shown in cyan, and DAPI is shown in yellow. Each row represents a subdivision of the IC. **(A–C)** Photomicrographs from the ICc. **(D–F)** Photomicrographs from the ICd. **(G–I)** Photomicrographs from the IClc. Scale bar = 100 μm.

We manually quantified each NPY mRNA across each age and IC subdivision (Figure 1 and Table 2). Figure 8 shows representative photomicrographs across the ICc, ICd, and IClc at 2–3 months of cells that express both NPY and GAD1 (white arrowheads) and GAD1 cells that do not express NPY mRNA (white arrows). Small cells had an average of 40.7 mRNA puncta in the ICc, 41.7 mRNA puncta in the ICd, and 35.5 mRNA puncta in the IClc (Table 1). Medium cells had an average of 80.3 mRNA puncta in the ICc, 92.7 mRNA puncta in the ICd, and 84.3 mRNA puncta in the IClc (Table 1). Large cells had an average of 173.7 mRNA puncta in the ICc, 222.5 mRNA puncta in the ICd, and 183.6 mRNA puncta in the IClc (Table 1).

**TABLE 2:** Neuropeptide Y (NPY) messenger ribonucleic acid (mRNA) across small, medium, and large inferior colliculus (IC) cells.

Case	Age (mo)	ICc small cell ave. #NPY mRNA	ICd small cell ave. #NPY mRNA	IClc small cell ave. #NPY mRNA	ICc medium cell ave. #NPY mRNA	ICd medium cell ave. #NPY mRNA	IClc medium cell ave. #NPY mRNA	ICc. large cell ave. #NPY mRNA	ICd large cell ave. #NPY mRNA	IClc large cell ave. #NPY mRNA
**2–3 months**										
R182	3	32	36.1	29.4	62.7	86.1	72.6	73.2	188.9	199.8
R183	3	27.5	18.7	18.7	72.4	88.2	77.2	199.2	233.3	121.3
R184	3	29.4	38.4	32.7	63	79.6	78.8	150.2	194.8	140
R185	3	44.7	45.5	43.1	104.8	115.4	107.7	183.2	278.2	277.2
R219	2	48.3	43.9	57	82.3	87.4	88.5	204.4	225	157
	–	40.7	41.7	35.5	80.3	92.7	84.3	173.74	222.5	183.6
**19 months**										
R180	19	52	49.3	51.3	103.9	98.5	96.1	217.4	239.4	173.4
R181	19	47.6	56.6	51	94.1	107.6	89.3	151.7	211.8	157.2
R213	19	40.8	40.4	29.9	77.9	82.2	72.6	151.3	223.5	119.8
R214	19	46.3	38	30	79.8	85.3	84.6	148.3	184.8	170.7
R215	19	40.5	51	n/a	86	84.1	77.2	163.3	211.9	190.1
	–	43.2	47.3	37.9	86.9	89.8	84.4	181.5	230.1	166.2
**24 months**										
R210	24	37	51.5	46	87.7	90.7	100.5	107.4	166.5	191.3
R211	24	35.6	47.6	40.2	76.9	98.6	103.8	176.3	253.5	257.9
R212	24	63.7	58.7	63.5	124.9	115.7	123.5	233.1	216.5	283.4
R216	24	19	55	64.7	82.9	102.3	131.6	218.3	313	195.5
R217	24	43.5	48.3	39.5	81.3	95	89.6	157.2	138.5	143.2
		38.1	49.1	48.9	86.4	99.1	109.8	183.7	183.7	212
**28 months**										
R176	28	31.6	34.9	35	74.5	76.7	65.3	141.7	168	130.2
R177	28	n/a	8	10	16.24	20.5	17.1	17	63.5	32
R178	28	48	31	n/a	67.3	63.1	63.4	127.5	173	197
R179	28	62.5	61.9	66.4	94.2	101.5	118.6	224	303.2	264.5
R209	28	57.4	64.5	46.1	104.8	121.7	119.5	449	n/a	n/a
		55.2	57.7	51.4	67.8	72.6	69	143.5	183.3	136.5

### 3.4 Expression of NPY mRNA in 19 months old FBN rats

**FIGURE 9:**
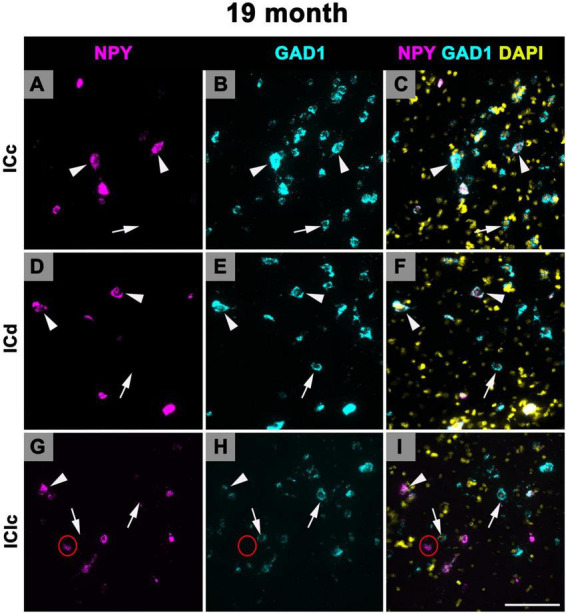
Structured illumination fluorescence images, taken at 0.2 μm steps, showing cells that co-express Neuropeptide Y (NPY) messenger ribonucleic acid (mRNA) and glutamate decarboxylase 1 (GAD1) mRNA (arrowheads) and cells that only express GAD1 mRNA (arrows) in an early middle age (19 months) animal. NPY mRNA is shown in magenta, GAD1 mRNA is shown in cyan, and DAPI is shown in yellow. Each row represents a subdivision of the IC. **(A–C)** Photomicrographs from the ICc. **(D–F)** Photomicrographs from the ICd. **(G–I)** Photomicrographs from the IClc. Red circle depicts a cell that is expressing NPY mRNA only. Scale bar = 100 μm.

Figure 9 shows representative photomicrographs across the ICc, ICd, and IClc at 19 months of cells that express both NPY and GAD1 (white arrowheads) and GAD1 cells that do not express NPY mRNA (white arrows). Small cells had an average of 43.2 mRNA puncta in the ICc, 47.3 mRNA puncta in the ICd, and 37.9 mRNA puncta in the IClc (Table 1). Medium cells had an average of 86.9 mRNA puncta in the ICc, 89.8 mRNA puncta in the ICd, and 84.4 mRNA puncta in the IClc (Table 1). Large cells had an average of 181.5 mRNA puncta in the ICc, 230.1 mRNA puncta in the ICd, and 166.2 mRNA puncta in the IClc (Table 1).

### 3.5 Expression of NPY mRNA in 24 months old FBN rats

**FIGURE 10:**
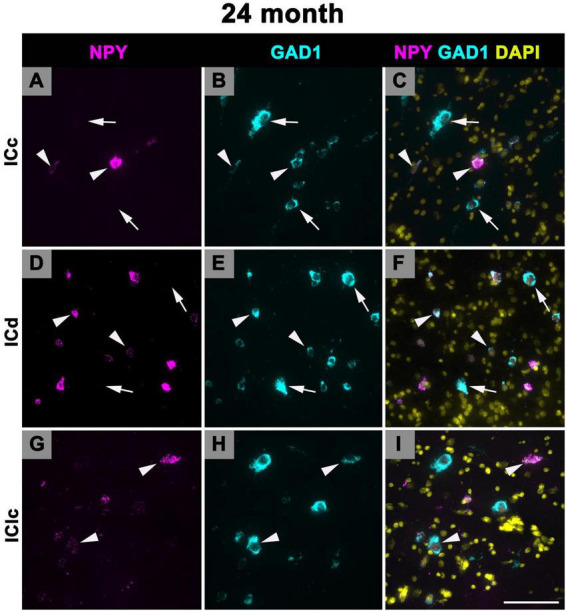
Structured illumination fluorescence images, taken at 0.2 μm steps, showing cells that co-express Neuropeptide Y (NPY) messenger ribonucleic acid (mRNA) and glutamate decarboxylase 1 (GAD1) mRNA (arrowheads) and cells that only express GAD1 mRNA (arrows) in a late middle age (24 months) animal. NPY mRNA is shown in magenta, GAD1 mRNA is shown in cyan, and DAPI is shown in yellow. Each row represents a subdivision of the IC. **(A–C)** Photomicrographs from the ICc. **(D–F)** Photomicrographs from the ICd. **(G–I)** Photomicrographs from the IClc. Scale bar = 100 μm.

Figure 10 shows representative photomicrographs across the ICc, ICd, and IClc at 24 months of cells that express both NPY and GAD1 (white arrowheads) and GAD1 cells that do not express NPY mRNA (white arrows). Small cells had an average of 38.1 mRNA puncta in the ICc, 49.1 mRNA puncta in the ICd, and 48.9 mRNA puncta in the IClc (Table 1). Medium cells had an average of 86.4 mRNA puncta in the ICc, 99.1 mRNA puncta in the ICd, and 109.8 mRNA puncta in the IClc (Table 1). Large cells had an average of 183.7 mRNA puncta in the ICc, 183.7 mRNA puncta in the ICd, and 212 mRNA puncta in the IClc (Table 1).

### 3.6 Expression of NPY mRNA in 28–29 months old FBN rats

**FIGURE 11:**
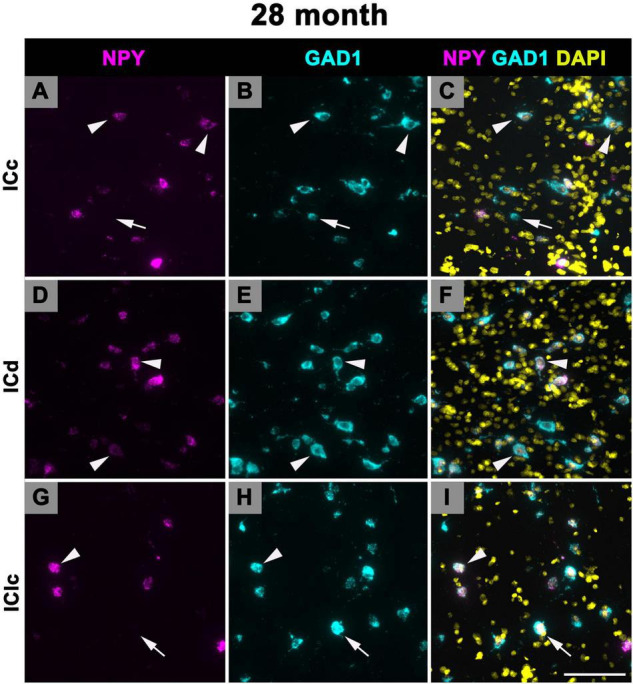
Structured illumination fluorescence images, taken at 0.2 μm steps, showing cells that co-express Neuropeptide Y (NPY) messenger ribonucleic acid (mRNA) and glutamate decarboxylase 1 (GAD1) mRNA (arrowheads) and cells that only express GAD1 mRNA (arrows) in an old (28 months) animal. NPY mRNA is shown in magenta, GAD1 mRNA is shown in cyan, and DAPI is shown in yellow. Each row represents a subdivision of the IC. **(A–C)** Photomicrographs from the ICc. **(D–F)** Photomicrographs from the ICd. **(G–I)** Photomicrographs from the IClc. Scale bar = 100 μm.

Figure 11 shows representative photomicrographs across the ICc, ICd, and IClc at 28 months of cells that express both NPY and GAD1 (white arrowheads) and GAD1 cells that do not express NPY mRNA (white arrows). Small cells had an average of 55.2 mRNA puncta in the ICc, 57.7 mRNA puncta in the ICd, and 51.4 mRNA puncta in the IClc (Table 1). Medium cells had an average of 67.8 mRNA puncta in the ICc, 72.6 mRNA puncta in the ICd, and 69 mRNA puncta in the IClc (Table 1). Large cells had an average of 143.5 mRNA puncta in the ICc, 183.3 mRNA puncta in the ICd, and 136.5 mRNA puncta in the IClc (Table 1).

### 3.7 NPY mRNA density per cellular profile area across age and IC subdivision

**FIGURE 12:**
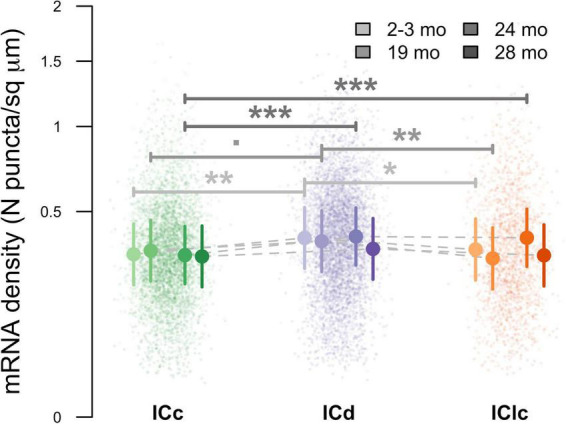
Variation in messenger ribonucleic acid (mRNA) density as a function of age and inferior colliculus (IC) subdivision [i.e., central (ICc), dorsal cortex (ICd), or lateral cortex (IClc)]. Raw data were log-transformed prior to analysis to improve distributional normality. Small circles indicate replicate measurements of individual animals. Large circles with error bars represent estimated marginal means within each age group and IC subdivision, and 95% confidence intervals around these means, respectively, as predicted by the mixed-effects regression model. For both small and large circles, darker colors indicated older ages, as shown in the legend. Horizontal bars indicate significant differences between ages within groups (= *p* < 0.1; *, *p* < 0.05; **, *p* < 0.01; ***, *p* < 0.001).

Lastly, pairwise comparisons between subdivisions at each age were computed to examine the density of NPY mRNA per cellular profile area (μm^2^). At 2–3 months ICd had a greater density of NPY mRNA than both ICc (**p = 0.0021) and IClc (*p = 0.0238; Figure 12). No significant difference in mRNA density was found between ICc and IClc at 2–3 months (*p* = 0.2938; Figure 12). At 19 months ICd had a greater density of NPY mRNA than IClc (**p = 0.0023; Figure 12). Although not quite reaching statistical significance, there was a trend showing greater mRNA density in ICd than ICc at 19 months (*p* = 0.0567). No significant difference in mRNA density was found between ICc and IClc at 19 months (*p* = 0.1072). At 24 months of age the density of NPY mRNA was significantly higher in the ICd than the ICc (***p = 0.0005; Figure 12), and higher in the IClc than the ICc (***p = 0.0009; Figure 12). No significance was found between ICd and IClc (*p* = 0.7854). There were no significant pairwise differences in NPY mRNA density among IC subdivisions at 28 months (all *p* ≥ 0.2352). Finally, pairwise comparisons between ages, within subdivisions, demonstrated no significant changes to NPY mRNA density due to age (Figure 12). We conclude that aging is not a factor that affects NPY mRNA density in the IC, though repeating this study with a larger sample size, and thus increased statistical power, may discern significant age related effects.

## 4 Discussion

The current study qualitatively and quantitatively describes NPY cell density and mRNA counts in the aging IC. Our findings demonstrate that NPY is expressed in all three subdivisions of the FBN rat IC, which agrees with previous studies conducted in other rat strains and mouse (Nakagawa et al., 1995; Silveira et al., 2020, 2023). We show that ∼38% of GABAergic IC cells produce NPY mRNA with the greatest proportion (> 60%) occurring in the ICd. Additionally, we demonstrate that 79% of NPY cells have medium sized profile areas. Perhaps the most interesting finding is that the ICd had a greater density of NPY mRNA per cellular profile area, as compared to the ICc and IClc, in our 2–3, 19, and 24 months old animals. However, at 28 months, there was no significant difference in NPY mRNA density between any of the IC subdivisions. Although we hypothesized that NPY mRNA is downregulated with age, our data did not find a significant reduction in mRNA due to aging. Thus, while inhibition declines with age, it appears that NPY driven inhibition may stay intact. NPY-expressing GABAergic neurons may represent a robust population that persists well into old age, suggesting that they are not the primary contributor to age-related loss of inhibition and central neural gain.

### 4.1 Technical considerations

The National Institute of Aging has recommended the FBN rat as a preferred model of aging because of its long median lifespan, as compared to other strains of mice and rats (Lipman et al., 1996; Lipman, 1997). As such, the FBN rat is commonly used in aging studies of the central auditory system (Caspary et al., 2008; Caspary and Llano, 2018; Cai et al., 2018; Robinson et al., 2019; Mafi et al., 2020, 2021, 2022; Kommajosyula et al., 2021). In this study we use four age groups. Our 2–3 months group equates to the standard “young” age group, when there are no hearing deficits, and our 28 months group is the standard “old” age group, when hearing loss is prevalent. Hearing deficits are uncommon in the 19 months middle age group, while hearing deficits are common in the 24 months middle age group (Cai et al., 2018). However, we acknowledge that a weakness in the study is that these rats did not undergo any physiological characterization or auditory brainstem responses, thus we ultimately do not know if our rats did or did not have any hearing loss at a given age.

As in our previous study, the current study does not rely on aldehyde fixation when extracting and sectioning the brain (Koehler et al., 2023). As mentioned in the Methods, when establishing profile area size for GABAergic cells in the IC for our smFISH studies, profile areas were log transformed in R to establish a quantile distribution that define our small (< 124 μm^2^), medium (124–411 μm^2^) and large (> 411 μm^2^) profile sizes. Previous studies classify medium-sized GABAergic IC cells as having profiles areas up to 300 μm^2^ (Beebe et al., 2016, 2018). Our higher top-end values (411 vs. 300 μm^2^) may be due to the lack of fixation and thus less overall shrinkage that is common in standard immunohistochemistry techniques. Also, the tissue for this study was sectioned at 12 μm which may result in a less accurate profile area than studies sectioning at 40–50 μm due to an increased z-axis to examine.

To label GABAergic IC cells we used a GAD1 mRNA probe for the FBN rat as we have previously had success with this gene and GAD1 is a commonly used marker to identify GABAergic cell populations (Dong et al., 2009; Koehler et al., 2023). As our smFISH multiplexing only utilized probes for NPY and GAD1 mRNA, we suggest that the small population of NPY cells we found that did not co-express GAD1, may express GAD2. The IC contains both GAD65- and GAD67- immunoreactive neurons, whose precursors are GAD2 and GAD1, respectively (Burianova et al., 2009; Harding et al., 2024). If NPY in the IC is only co-expressed in GABAergic cells we presume that the cells expressing only NPY mRNA may express genes for GAD2 and therefore may be GABAergic as well. As GABAergic cells throughout the brain may express GAD1 and GAD2, our “NPY-only” cells may simply be a result of the smFISH technique not labeling all mRNA. However, further experiments exploring this distinction would be needed.

### 4.2 Comparison to previous studies

Our data are consistent with knowledge that NPY in the brain is co-expressed by GABAergic cell populations (Sperk et al., 2007; Ramamoorthy et al., 2011; Aoki and Pickel, 1990; Lall and Biello, 2003; Parker et al., 1998; Kozicz and Lázár, 2001). This relationship is also apparent in the IC as GABAergic cells co-express NPY (Silveira et al., 2020, 2023; Anair et al., 2022). Specifically, previous studies and the current study have focused on the subset of GABAergic principal neurons that co-express NPY (Silveira et al., 2020, 2023). In these studies of the mouse, roughly one-third of the GABAergic IC cells also expressed NPY (Silveira et al., 2020). Our data are consistent with these studies as we found ∼38% of FBN rat GABAergic IC cells also express NPY. Unlike GABAergic IC neurons which are commonly dispersed evenly throughout much of the IC (Oliver et al., 1994; Winer et al., 1995, 1996; Oliver, 2005), we found a non-uniform distribution of NPY expressing cells in the IC such that the ICd had the largest proportion of NPY cells and the largest population of GABAergic cells co-expressing NPY. This finding agrees with Nakagawa et al., 1995, as they also report NPY expression being most robust in the dorsal IC.

GABAergic IC cells are classically characterized as small, medium, or large based on soma size (Ono et al., 2005; Ito et al., 2009; Altschuler et al., 2008; Roberts and Ribak, 1987a,b; Oliver, 2005; Beebe et al., 2016; Koehler et al., 2023). In the current study we defined the vast majority (∼80%) of NPY positive cells as medium-sized. GABAergic cell size has been linked to the intrinsic patterns and projections of the cells (Ono et al., 2005; Ito et al., 2009; Geis and Borst, 2013; Beebe et al., 2016, 2018). Medium-sized GABAergic neurons participate in several long range projections, including outputs to the medial geniculate body (MG), commissural projections to the contralateral IC, and projections to the superior colliculus (SC) (Winer et al., 1996; Oliver, 2005; Mellott et al., 2018, 2019). In the lemniscal IC, cell types are commonly categorized as either disk/flat or stellate/less-flat (Oliver, 2005; Mellott et al., 2014b; Beebe et al., 2016). Also, a more recent study identified basket cell morphologies with distinct physiological profiles in the lemniscal IC (Wallace et al., 2021). Very little is understood about the morphological cell types throughout the non-lemniscal IC. In Silveira (’20) it was demonstrated in the mouse that all NPY cells they had recorded from maintained a stellate morphology regardless of IC subdivision. If NPY cell type is consistent across species, we would suggest that NPY inhibition in the FBN rat is largely driven by medium-sized stellate cells. Previous studies have demonstrated that GABAergic cells can be categorized by the presence of perineuronal nets and/or dense VGlut2-containing inputs (Ito et al., 2009; Beebe et al., 2016, 2018). The current study suggests that NPY cells are “medium-sized and thus may or may not be surrounded by perineuronal nets and/or VGlut2 rings (Beebe et al., 2016, 2018). However, these previous studies used fixed tissue to evaluate their cells size, while in the current approach the cells are unfixed at tissue extraction which results in slight larger average due to a lack of shrinkage (Koehler et al., 2023). It will be interesting for further studies co-labeling NPY, VGlut2, and perineural nets to illuminate these points, and thus provide a greater functional context of IC NPY cells.

A major goal of the current study was to determine if NPY mRNA was downregulated in the aging IC of the FBN rat in the same manner as our previous report on GAD1 (Koehler et al., 2023). Briefly, in our previous work GAD1 mRNA was downregulated with age in smaller GABAergic cells in the lower frequency region, upregulated in larger GABAergic cells, and generally unchanged in medium sized cells between young and old age groups (Koehler et al., 2023). As we found NPY to be most commonly expressed in medium sized cells, collectively it appears that medium sized GABAergic cells in the IC do not undergo robust age-related changed to their mRNA for major inhibitory neurotransmitters. In the current study, although fewer in number (∼20%) NPY mRNA in larger and smaller cells was also unchanged with age. We conclude that it is likely that an individual GABAergic cell can downregulate mRNA for one inhibitory gene (GAD1) while another (NPY) is unaffected by age.

### 4.3 Functional significance

It is only recently that we have gained a better understanding of the role of NPY in the IC as it has been demonstrated that NPY can inhibit local recurrent excitation (via the Y_1_ receptor) (Silveira et al., 2023). Additionally, NPY IC cells may provide lateral inhibition through wide-spread heterotopic commissural inputs (Anair et al., 2022). As NPY cell populations are found in each major IC subdivision, one could expect that functional properties persist from lemniscal to non-lemniscal circuits. Given that the non-lemniscal IC plays a large role in the integration of multisensory inputs and receives robust descending cortical inputs, it will be critical for future studies to determine the functional role of NPY across these diverse circuits.

Perhaps the most interesting finding from the current study is that NPY mRNA expression is not affected by age in any IC subdivision. It is well-known that GABAergic inhibition is downregulated in the aging IC (Caspary et al., 2008; Syka, 2020). This initial downregulation of GABAergic inhibition is commonly associated with mechanisms to maintain homeostatic balance during peripheral deafferentation (Kotak et al., 2005, Caspary et al., 2008, Rabang et al., 2012; Resnik and Polley, 2017; Parthasarathy and Kujawa, 2018). However, the continued loss of GABAergic inhibition can lead to many deficits including degraded temporal and binaural coding in speech perception, and the over-amplification of sound-evoked neural responses known as “central gain” (Walton et al., 1998, 2002; Frisina and Rajan, 2005 Kotak et al., 2005, Caspary et al., 2008, Walton, 2010; Parthasarathy and Bartlett, 2011 Rabang et al., 2012; Auerbach et al., 2014, 2019; Resnik and Polley, 2017; Parthasarathy and Kujawa, 2018). These mechanisms may be centrally generated and lead to hyperacusis and tinnitus (Auerbach et al., 2014, 2019). We conclude that NPY inhibition in the aging IC likely plays a very distinctive role from GABAergic inhibition. Studies of NPY across the brain suggest that NPY may help preserve function in aging neurons.

Our results suggest that NPY-mediated inhibition in the IC may not be a major contributor to the onset of presbycusis. Thus, it is possible that the functional roles of NPY mentioned above are intact and/or unaffected by aging. Studies of NPY function in the brain heavily imply that NPY may serve an anti-aging role by employing neuroprotective effects, decreasing excitotoxicity, regulating calcium homeostasis, protecting against oxidative stress and mitochondrial dysfunction, and reducing neuroinflammation (see reviews: Botelho and Cavadas, 2015; Li et al., 2020; Chen et al., 2019; Duarte-Neves et al., 2016). Particularly in the hippocampus, studies have demonstrated that NPY prevents intracellular oxidative stress and activates pathways involved in neuroprotection (dos Santos et al., 2013). Additionally, NPY mediates the production of neurotrophins such as brain derived neurotrophic factor (BDNF) and nerve growth factor (NGF) (Croce et al., 2011, 2013; Angelucci et al., 2014). There are noteworthy similarities in NPY expression and function across the IC and hippocampus, including the co-release of NPY and GABA and NPY’s widespread effects on glutamatergic neurotransmission via the Y1 receptor (Sperk et al., 2007; Silveira et al., 2020, 2023). Determining whether various IC functions are preserved via NPY-mediated mechanisms will provide more clarity regarding the aging central auditory system.

The current study indicates that NPY mRNA production is preserved in the aging inferior colliculus, which may signify that NPY helps to maintain function well into old age. We have demonstrated that transcription of NPY mRNA remains intact with age, but further studies are required to determine whether NPY protein translation is also unaffected by aging. Furthermore, future studies are needed to explore the juxtaposition of NPY versus GABA changes with age. Perhaps the persistence of NPY levels in the aging IC serves to provide compensation of function while GABAergic inhibition is being reduced over the lifespan. Additionally, it remains to be determined whether NPY synapses and their postsynaptic contacts are altered with age, similar to GABA (Helfert et al., 1999). And the age-related changes of the NPY Y1 receptor remains, to our knowledge, virtually untouched. Ultimately, the broad role of NPY on hearing at any age requires further exploration.

## Data Availability

The original contributions presented in this study are included in this article/Supplementary material, further inquiries can be directed to the corresponding author.

## References

[B1] Altschuler R. A. Tong L. Holt A. G. Oliver D. L. (2008). Immunolocalization of vesicular glutamate transporters 1 and 2 in the rat inferior colliculus. *Neuroscience* 154 226–232. 10.1016/j.neuroscience.2008.03.036 18436385 PMC2574917

[B2] Anair J. D. Silveira M. A. Mirjalili P. Beebe N. L. Schofield B. R. Roberts M. T. (2022). Inhibitory NPY neurons provide a large and heterotopic commissural projection in the inferior colliculus. *Front. Neural Circuits* 16:871924. 10.3389/fncir.2022.871924 35693026 PMC9178209

[B3] Angelucci F. Gelfo F. Fiore M. Croce N. Mathé A. A. Bernardini S. (2014). The effect of neuropeptide Y on cell survival and neurotrophin expression in in-vitro models of Alzheimer’s disease. *Can. J. Physiol. Pharmacol.* 92 621–630. 10.1139/cjpp-2014-0099 25026432

[B4] Aoki C. Pickel V. M. (1990). Neuropeptide Y in cortex and striatum. Ultrastructural distribution and coexistence with classical neurotransmitters and neuropeptides. *Ann. N. Y. Acad. Sci.* 611 186–205. 10.1111/j.1749-6632.1990.tb48931.x 2174219

[B5] Auerbach B. D. Radziwon K. Salvi R. (2019). Testing the central gain model: Loudness growth correlates with central auditory gain enhancement in a rodent model of hyperacusis. *Neuroscience* 407 93–107. 10.1016/j.neuroscience.2018.09.036 30292765 PMC8792806

[B6] Auerbach B. D. Rodrigues P. V. Salvi R. J. (2014). Central gain control in tinnitus and hyperacusis. *Front. Neurol.* 5:206. 10.3389/fneur.2014.00206 25386157 PMC4208401

[B7] Bates D. Mächler M. Bolker B. Walker S. (2015). Fitting linear mixed-effects models using lme4. *J. Statist. Softw.* 67 1–48. 10.18637/jss.v067.i01

[B8] Beebe N. L. Mellott J. G. Schofield B. R. (2018). Inhibitory projections from the inferior colliculus to the medial geniculate body originate from four subtypes of GABAergic cells. *eNeuro* 5:ENEURO.0406-18.2018. 10.1523/ENEURO.0406-18.2018 30456294 PMC6240760

[B9] Beebe N. L. Young J. W. Mellott J. G. Schofield B. R. (2016). Extracellular molecular markers and soma size of inhibitory neurons: Evidence for four subtypes of GABAergic cells in the inferior colliculus. *J. Neurosci.* 36 3988–3999. 10.1523/JNEUROSCI.0217-16.2016 27053206 PMC4821910

[B10] Benjamini Y. Hochberg Y. (1995). Controlling the false discovery rate: A practical and powerful approach to multiple testing. *J. R. Stat. Soc. Ser. B* 125 289–300. 10.1111/j.2517-6161.1995.tb02031.x

[B11] Botelho M. Cavadas C. (2015). Neuropeptide Y: An anti-aging player? *Trends Neurosci.* 38 701–711. 10.1016/j.tins.2015.08.012 26549884

[B12] Burianova J. Ouda L. Profant O. Syka J. (2009). Age-related changes in GAD levels in the central auditory system of the rat. *Exp. Gerontol.* 44 161–169. 10.1016/j.exger.2008.09.012 18930128

[B13] Cai R. Kalappa B. I. Brozoski T. J. Ling L. L. Caspary D. M. (2014). Is GABA neurotransmission enhanced in auditory thalamus relative to inferior colliculus? *J. Neurophysiol.* 111 229–238. 10.1152/jn.00556.2013 24155003 PMC3921384

[B14] Cai R. Montgomery S. C. Graves K. A. Caspary D. M. Cox B. C. (2018). The FBN rat model of aging: Investigation of ABR waveforms and ribbon synapse changes. *Neurobiol. Aging* 62 53–63. 10.1016/j.neurobiolaging.2017.09.034 29107847 PMC5743589

[B15] Caspary D. M. Ling L. Turner J. G. Hughes L. F. (2008). Inhibitory neurotransmission, plasticity and aging in the mammalian central auditory system. *J. Exp. Biol.* 211(Pt 11), 1781–1791. 10.1242/jeb.013581 18490394 PMC2409121

[B16] Caspary D. M. Llano D. A. (2018). “Aging process in the subcortical auditory system,” in *The Oxford handbook of the auditory brainstem*, ed. Kandler K. (Oxford: Oxford Press).

[B17] Caspary D. M. Milbrandt J. C. Helfert R. H. (1995). Central auditory aging: Gaba changes in the inferior colliculus. *Exp. Gerontol.* 30 349–360. 10.1016/0531-5565(94)00052-5 7556513

[B18] Caspary D. M. Raza A. Lawhorn Armour B. A. Pippin J. Arnerić S. P. (1990). Immunocytochemical and neurochemical evidence for age-related loss of GABA in the inferior colliculus: Implications for neural presbycusis. *J. Neurosci.* 10 2363–2372. 10.1523/JNEUROSCI.10-07-02363.1990 1973948 PMC6570369

[B19] Chen X. Y. Du Y. F. Chen L. (2019). Neuropeptides exert neuroprotective effects in Alzheimer’s disease. *Front. Mol. Neurosci.* 11:493. 10.3389/fnmol.2018.00493 30687008 PMC6336706

[B20] Croce N. Dinallo V. Ricci V. Federici G. Caltagirone C. Bernardini S. (2011). Neuroprotective effect of neuropeptide Y against β-amyloid 25-35 toxicity in SH-SY5Y neuroblastoma cells is associated with increased neurotrophin production. *Neurodegener. Dis.* 8 300–309. 10.1159/000323468 21346312

[B21] Croce N. Gelfo F. Ciotti M. T. Federici G. Caltagirone C. Bernardini S. (2013). NPY modulates miR-30a-5p and BDNF in opposite direction in an in vitro model of Alzheimer disease: A possible role in neuroprotection? *Mol. Cell Biochem.* 376 189–195. 10.1007/s11010-013-1567-0 23358924

[B22] Dong S. Mulders W. H. Rodger J. Robertson D. (2009). Changes in neuronal activity and gene expression in guinea-pig auditory brainstem after unilateral partial hearing loss. *Neuroscience* 159 1164–1174. 10.1016/j.neuroscience.2009.01.043 19356697

[B23] dos Santos V. V. Santos D. B. Lach G. Rodrigues A. L. Farina M. De Lima T. C. (2013). Neuropeptide Y (NPY) prevents depressive-like behavior, spatial memory deficits and oxidative stress following amyloid-β (Aβ(1-40)) administration in mice. *Behav. Brain Res.* 244 107–115. 10.1016/j.bbr.2013.01.039 23396168

[B24] Drotos A. C. Roberts M. T. (2024). Identifying neuron types and circuit mechanisms in the auditory midbrain. *Hear. Res.* 442:108938. 10.1016/j.heares.2023.108938 38141518 PMC11000261

[B25] Duarte-Neves J. Pereira de Almeida L. Cavadas C. (2016). Neuropeptide Y (NPY) as a therapeutic target for neurodegenerative diseases. *Neurobiol. Dis.* 95 210–224. 10.1016/j.nbd.2016.07.022 27461050

[B26] Edvinsson L. Emson P. McCulloch J. Tatemoto K. Uddman R. (1983). Neuropeptide Y: Cerebrovascular innervation and vasomotor effects in the cat. *Neurosci. Lett.* 43 79–84. 10.1016/0304-3940(83)90132-5 6689442

[B27] Faye-Lund H. Osen K. K. (1985). Anatomy of the inferior colliculus in rat. *Anat. Embryol.* 171 1–20. 10.1007/BF00319050 3985354

[B28] Frisina R. D. (2001). Subcortical neural coding mechanisms for auditory temporal processing. *Hear. Res.* 158 1–27. 10.1016/s0378-5955(01)00296-9 11506933

[B29] Frisina R. D. Rajan R. (2005). “Inferior colliculus: Aging and plasticity,” in *The inferior colliculus*, eds Winer J. A. Schreiner C. E. (New York, NY: Springer), 559–584.

[B30] Geis H. R. Borst J. G. (2013). Intracellular responses to frequency modulated tones in the dorsal cortex of the mouse inferior colliculus. *Front. Neural Circuits* 7:7. 10.3389/fncir.2013.00007 23386812 PMC3560375

[B31] Goman A. M. Lin F. R. (2016). Prevalence of hearing loss by severity in the United States. *Am. J. Public Health* 106 1820–1822. 10.2105/AJPH.2016.303299 27552261 PMC5024365

[B32] González-Hernández T. Mantolán-Sarmiento B. González-González B. Pérez-González H. (1996). Sources of GABAergic input to the inferior colliculus of the rat. *J. Comp. Neurol.* 372 309–326. 10.1002/(SICI)1096-9861(19960819)372:2<309::AID-CNE11<3.0.CO;2-E 8863133

[B33] Gøtzsche C. R. Woldbye D. P. (2016). The role of NPY in learning and memory. *Neuropeptides* 55 79–89. 10.1016/j.npep.2015.09.010 26454711

[B34] Goyer D. Silveira M. A. George A. P. Beebe N. L. Edelbrock R. M. Malinski P. T. (2019). A novel class of inferior colliculus principal neurons labeled in vasoactive intestinal peptide-Cre mice. *Elife* 8:e43770. 10.7554/eLife.43770 30998185 PMC6516826

[B35] Gutiérrez A. Khan Z. U. Morris S. J. De Blas A. L. (1994). Age-related decrease of GABAA receptor subunits and glutamic acid decarboxylase in the rat inferior colliculus. *J. Neurosci.* 14 7469–7477. 10.1523/JNEUROSCI.14-12-07469.1994 7996188 PMC6576900

[B36] Haile L. M. Kamenov K. Briant P. S. Orji A. U. Steinmetz J. D. Abdoli A. (2021). Hearing loss prevalence and years lived with disability, 1990–2019: Findings from the Global Burden of Disease Study 2019. *Lancet* 397 996–1009. 10.1016/S0140-6736(21)00516-X 33714390 PMC7960691

[B37] Harding E. K. Zhang Z. Canet-Pons J. Stokes-Heck S. Trang T. Zamponi G. W. (2024). Expression of GAD2 in excitatory neurons projecting from the ventrolateral periaqueductal gray to the locus coeruleus. *iScience* 27:109972. 10.1016/j.isci.2024.109972 38868198 PMC11166693

[B38] Harrison X. A. Donaldson L. Correa-Cano M. E. Evans J. Fisher D. N. Goodwin C. E. D. (2018). A brief introduction to mixed effects modelling and multi-model inference in ecology. *PeerJ* 6:e4794. 10.7717/peerj.4794 29844961 PMC5970551

[B39] Helfert R. H. Sommer T. J. Meeks J. Hofstetter P. Hughes L. F. (1999). Age-related synaptic changes in the central nucleus of the inferior colliculus of Fischer-344 rats. *J. Comp. Neurol.* 406 285–298. 10.1002/(SICI)1096-9861(19990412)406:3<285::AID-CNE1<3.0.CO;2-P 10102497

[B40] Ibrahim B. A. Llano D. A. (2019). Aging and central auditory disinhibition: Is it a reflection of homeostatic downregulation or metabolic vulnerability? *Brain Sci.* 9:351. 10.3390/brainsci9120351 31805729 PMC6955996

[B41] Ito T. Bishop D. C. Oliver D. L. (2009). Two classes of GABAergic neurons in the inferior colliculus. *J. Neurosci.* 29 13860–13869. 10.1523/JNEUROSCI.3454-09.2009 19889997 PMC2814801

[B42] Knipper M. Singer W. Schwabe K. Hagberg G. E. Li Hegner Y. Rüttiger L. (2022). Disturbed balance of inhibitory signaling links hearing loss and cognition. *Front. Neural Circuits* 15:785603. 10.3389/fncir.2021.785603 35069123 PMC8770933

[B43] Koehler C. C. Almassri L. S. Tokar N. Mafi A. M. O’Hara M. J. Young J. W. (2023). Age-related changes of GAD1 mRNA expression in the central inferior colliculus. *Transl. Med. Aging* 7 20–32. 10.1016/j.tma.2023.04.001 38111912 PMC10727507

[B44] Kommajosyula S. P. Bartlett E. L. Cai R. Ling L. Caspary D. M. (2021). Corticothalamic projections deliver enhanced responses to medial geniculate body as a function of the temporal reliability of the stimulus. *J. Physiol.* 599 5465–5484. 10.1113/JP282321 34783016 PMC10630908

[B45] Kotak V. C. Fujisawa S. Lee F. A. Karthikeyan O. Aoki C. Sanes D. H. (2005). Hearing loss raises excitability in the auditory cortex. *J. Neurosci.* 25 3908–3918. 10.1523/JNEUROSCI.5169-04.2005 15829643 PMC1764814

[B46] Kozicz T. Lázár G. (2001). Colocalization of GABA, enkephalin and neuropeptide Y in the tectum of the green frog Rana esculenta. *Peptides* 22 1071–1077. 10.1016/s0196-9781(01)00430-2 11445236

[B47] Kuznetsova A. Brockhoff P. B. Christensen R. H. (2017). lmerTest package: Tests in linear mixed effects models. *J. Statist. Softw.* 82 1–26. 10.18637/jss.v082.i13

[B48] Lall G. S. Biello S. M. (2003). Neuropeptide Y. *Neuroscience* 120 915–921. 10.1016/s0306-4522(03)00396-8 12927198

[B49] Lenth R. V. (2024). *emmeans: Estimated Marginal Means, aka Least-Squares Means. R package version 1103.* Available online at: https://CRAN.R-project.org/package=emmeans (accessed February 2025).

[B50] Li C. Luo T. Cheng Y. Liu S. Qiao L. Wu X. (2020). The effects of IVIg therapy on serum levels of neuropeptide Y and cytokines in Guillain-Barré syndrome. *Neurol. Sci.* 41 295–303. 10.1007/s10072-019-04063-3 31494821

[B51] Li-Korotky H. S. (2012). Age-related hearing loss: Quality of care for quality of life. *Gerontologist* 52 265–271. 10.1093/geront/gnr159 22383543

[B52] Lipman R. D. (1997). Pathobiology of aging rodents: Inbred and hybrid models. *Exp. Gerontol.* 32 215–228. 10.1016/s0531-5565(96)00037-x 9088918

[B53] Lipman R. D. Chrisp C. E. Hazzard D. G. Bronson R. T. (1996). Pathologic characterization of brown Norway, brown Norway x Fischer 344, and Fischer 344 x brown Norway rats with relation to age. *J. Gerontol. A Biol. Sci. Med. Sci.* 51 B54–B59. 10.1093/gerona/51a.1.b54 8548501 PMC7110307

[B54] Loftus W. C. Malmierca M. S. Bishop D. C. Oliver D. L. (2008). The cytoarchitecture of the inferior colliculus revisited: A common organization of the lateral cortex in rat and cat. *Neuroscience* 154 196–205. 10.1016/j.neuroscience.2008.01.019 18313229 PMC2562950

[B55] Mafi A. M. Hofer L. N. Russ M. G. Young J. W. Mellott J. G. (2020). The density of perineuronal nets increases with age in the inferior colliculus in the Fischer Brown Norway rat. *Front. Aging Neurosci.* 12:27. 10.3389/fnagi.2020.00027 32116654 PMC7026493

[B56] Mafi A. M. Russ M. G. Hofer L. N. Pham V. Q. Young J. W. Mellott J. G. (2021). Inferior collicular cells that project to the auditory thalamus are increasingly surrounded by perineuronal nets with age. *Neurobiol. Aging* 105 1–15. 10.1016/j.neurobiolaging.2021.04.001 34004491 PMC8338758

[B57] Mafi A. M. Tokar N. Russ M. G. Barat O. Mellott J. G. (2022). Age-related ultrastructural changes in the lateral cortex of the inferior colliculus. *Neurobiol. Aging* 120 43–59. 10.1016/j.neurobiolaging.2022.08.007 36116395 PMC10276896

[B58] Man J. Chen H. Zhang T. Yin X. Yang X. Lu M. (2021). Global, regional, and national burden of age-related hearing loss from 1990 to 2019. *Aging (Albany NY)* 13 25944–25959. 10.18632/aging.203782 34910687 PMC8751586

[B59] Mellott J. G. Beebe N. L. Schofield B. R. (2018). GABAergic and non-GABAergic projections to the superior colliculus from the auditory brainstem. *Brain Struct. Funct.* 223 1923–1936. 10.1007/s00429-017-1599-4 29302743 PMC5886796

[B60] Mellott J. G. Beebe N. L. Schofield B. R. (2019). Bilateral projections to the thalamus from individual neurons in the inferior colliculus. *J. Comp. Neurol.* 527 1118–1126. 10.1002/cne.24600 30536721 PMC6368862

[B61] Mellott J. G. Dhar M. Mafi A. Tokar N. Winters B. D. (2022). Tonotopic distribution and inferior colliculus projection pattern of inhibitory and excitatory cell types in the lateral superior olive of Mongolian gerbils. *J. Comp. Neurol.* 530 506–517. 10.1002/cne.25226 34338321 PMC8716415

[B62] Mellott J. G. Foster N. L. Nakamoto K. T. Motts S. D. Schofield B. R. (2014a). Distribution of GABAergic cells in the inferior colliculus that project to the thalamus. *Front. Neuroanat.* 8:17. 10.3389/fnana.2014.00017 24744703 PMC3978371

[B63] Mellott J. G. Foster N. L. Ohl A. P. Schofield B. R. (2014b). Excitatory and inhibitory projections in parallel pathways from the inferior colliculus to the auditory thalamus. *Front. Neuroanat.* 8:124. 10.3389/fnana.2014.00124 25414646 PMC4220731

[B64] Merchán M. Aguilar L. A. Lopez-Poveda E. A. Malmierca M. S. (2005). The inferior colliculus of the rat: Quantitative immunocytochemical study of GABA and glycine. *Neuroscience* 136 907–925. 10.1016/j.neuroscience.2004.12.030 16344160

[B65] Milbrandt J. C. Albin R. L. Caspary D. M. (1994). Age-related decrease in GABAB receptor binding in the Fischer 344 rat inferior colliculus. *Neurobiol. Aging* 15 699–703. 10.1016/0197-4580(94)90051-5 7891824

[B66] Milbrandt J. C. Albin R. L. Turgeon S. M. Caspary D. M. (1996). GABAA receptor binding in the aging rat inferior colliculus. *Neuroscience* 73 449–458. 10.1016/0306-4522(96)00050-4 8783261

[B67] Milbrandt J. C. Hunter C. Caspary D. M. (1997). Alterations of GABAA receptor subunit mRNA levels in the aging Fischer 344 rat inferior colliculus. *J. Comp. Neurol.* 379 455–465. 10.1002/(sici)1096-9861(19970317)379:3&It;455::aid-cne10>3.0.co;2-f 9067836

[B68] Nakagawa H. Ikeda M. Houtani T. Ueyama T. Baba K. Kondoh A. (1995). Immunohistochemical evidence for enkephalin and neuropeptide Y in rat inferior colliculus neurons that provide ascending or commissural fibers. *Brain Res.* 690 236–240. 10.1016/0006-8993(95)00593-f 8535842

[B69] Oliver D. L. (2005). “Neuronal organization in the inferior colliculus,” in *The inferior colliculus*, eds Winer J. A. Schreiner C. E. (New York, NY: Springer).

[B70] Oliver D. L. Winer J. A. Beckius G. E. Saint Marie R. L. (1994). Morphology of GABAergic neurons in the inferior colliculus of the cat. *J. Comp. Neurol.* 340 27–42. 10.1002/cne.903400104 7909821

[B71] Ono M. Oliver D. L. (2014). The balance of excitatory and inhibitory synaptic inputs for coding sound location. *J. Neurosci.* 34 3779–3792. 10.1523/JNEUROSCI.2954-13.2014 24599475 PMC3942590

[B72] Ono M. Yanagawa Y. Koyano K. (2005). GABAergic neurons in inferior colliculus of the GAD67-GFP knock-in mouse: Electrophysiological and morphological properties. *Neurosci. Res.* 51 475–492. 10.1016/j.neures.2004.12.019 15740810

[B73] Ouda L. Profant O. Syka J. (2015). Age-related changes in the central auditory system. *Cell Tissue Res.* 361 337–358. 10.1007/s00441-014-2107-2 25630878

[B74] Ouda L. Syka J. (2012). Immunocytochemical profiles of inferior colliculus neurons in the rat and their changes with aging. *Front. Neural Circuits* 6:68. 10.3389/fncir.2012.00068 23049499 PMC3448074

[B75] Pal I. Paltati C. R. B. Kaur C. Saini S. Kumar P. Jacob T. G. (2019). Morphological and neurochemical changes in GABAergic neurons of the aging human inferior colliculus. *Hear. Res.* 377 318–329. 10.1016/j.heares.2019.02.005 30878270

[B76] Palombi P. S. Caspary D. M. (1996a). GABA inputs control discharge rate primarily within frequency receptive fields of inferior colliculus neurons. *J. Neurophysiol.* 75 2211–2219. 10.1152/jn.1996.75.6.2211 8793735

[B77] Palombi P. S. Caspary D. M. (1996b). Responses of young and aged Fischer 344 rat inferior colliculus neurons to binaural tonal stimuli. *Hear. Res.* 100 59–67. 10.1016/0378-5955(96)00113-x 8922980

[B78] Parker D. Söderberg C. Zotova E. Shupliakov O. Langel U. Bartfai T. (1998). Co-localized neuropeptide Y and GABA have complementary presynaptic effects on sensory synaptic transmission. *Eur. J. Neurosci.* 10 2856–2870. 10.1111/j.1460-9568.1998.00295.x 9758155

[B79] Parthasarathy A. Bartlett E. L. (2011). Age-related auditory deficits in temporal processing in F-344 rats. *Neuroscience* 192 619–630. 10.1016/j.neuroscience.2011.06.042 21723376

[B80] Parthasarathy A. Kujawa S. G. (2018). Synaptopathy in the aging cochlea: Characterizing early-neural deficits in auditory temporal envelope processing. *J. Neurosci.* 38 7108–7119. 10.1523/JNEUROSCI.3240-17.2018 29976623 PMC6596096

[B81] Paxinos G. Watson C. (1998). *The rat brain in stereotaxic coordinates.* San Diego, CA: Academic Press.

[B82] Peruzzi D. Bartlett E. Smith P. H. Oliver D. L. (1997). A monosynaptic GABAergic input from the inferior colliculus to the medial geniculate body in rat. *J. Neurosci.* 17 3766–3777. 10.1523/JNEUROSCI.17-10-03766.1997 9133396 PMC6573711

[B83] Pollak G. D. Xie R. Gittelman J. X. Andoni S. Li N. (2011). The dominance of inhibition in the inferior colliculus. *Hear. Res.* 274 27–39. 10.1016/j.heares.2010.05.010 20685288 PMC3762690

[B84] R Core Team (2025). *R: A language and environment for statistical computing. 4.2.3, “Shortstop Beagle”.* Vienna: R Foundation for Statistical Computing.

[B85] Rabang C. F. Parthasarathy A. Venkataraman Y. Fisher Z. L. Gardner S. M. Bartlett E. L. (2012). A computational model of inferior colliculus responses to amplitude modulated sounds in young and aged rats. *Front. Neural Circuits* 6:77. 10.3389/fncir.2012.00077 23129994 PMC3487458

[B86] Ramamoorthy P. Wang Q. Whim M. D. (2011). Cell type-dependent trafficking of neuropeptide Y-containing dense core granules in CNS neurons. *J. Neurosci.* 31 14783–14788. 10.1523/JNEUROSCI.2933-11.2011 21994394 PMC3342306

[B87] Ramamoorthy P. Whim M. D. (2008). Trafficking and fusion of neuropeptide Y-containing dense-core granules in astrocytes. *J. Neurosci.* 28 13815–13827. 10.1523/JNEUROSCI.5361-07.2008 19091972 PMC2635891

[B88] Raza A. Milbrandt J. C. Arneric S. P. Caspary D. M. (1994). Age-related changes in brainstem auditory neurotransmitters: Measures of GABA and acetylcholine function. *Hear. Res.* 77 221–230. 10.1016/0378-5955(94)90270-4 7928735

[B89] Resnik J. Polley D. B. (2017). Fast-spiking GABA circuit dynamics in the auditory cortex predict recovery of sensory processing following peripheral nerve damage. *Elife* 6:e21452. 10.7554/eLife.21452 28323619 PMC5378474

[B90] Roberts R. C. Ribak C. E. (1987a). An electron microscopic study of GABAergic neurons and terminals in the central nucleus of the inferior colliculus of the rat. *J. Neurocytol.* 16 333–345. 10.1007/BF01611345 3302119

[B91] Roberts R. C. Ribak C. E. (1987b). GABAergic neurons and axon terminals in the brainstem auditory nuclei of the gerbil. *J. Comp. Neurol.* 258 267–280. 10.1002/cne.902580207 3584540

[B92] Robinson L. C. Barat O. Mellott J. G. (2019). GABAergic and glutamatergic cells in the inferior colliculus dynamically express the GABAAR γ1 subunit during aging. *Neurobiol. Aging* 80 99–110. 10.1016/j.neurobiolaging.2019.04.007 31112831

[B93] Rumschlag J. A. McClaskey C. M. Dias J. W. Kerouac L. B. Noble K. V. Panganiban C. (2022). Age-related central gain with degraded neural synchrony in the auditory brainstem of mice and humans. *Neurobiol. Aging* 115 50–59. 10.1016/j.neurobiolaging.2022.03.014 35468552 PMC9153923

[B94] Salehi P. Nelson C. N. Chen Y. Lei D. Crish S. D. Nelson J. (2018). Detection of single mRNAs in individual cells of the auditory system. *Hear. Res.* 367 88–96. 10.1016/j.heares.2018.07.008 30071403

[B95] Schofield B. R. (2010). “Structural organization of the descending auditory pathway,” in *The Oxford handbook of auditory neuroscience*, eds Rees A. Palmer A. (Oxford: Oxford University Press).

[B96] Schofield B. R. Beebe N. L. (2019). Subtypes of GABAergic cells in the inferior colliculus. *Hear. Res.* 376 1–10. 10.1016/j.heares.2018.10.001 30314930 PMC6447491

[B97] Silveira M. A. Anair J. D. Beebe N. L. Mirjalili P. Schofield B. R. Roberts M. T. (2020). Neuropeptide Y expression defines a novel class of GABAergic projection neuron in the inferior colliculus. *J. Neurosci.* 40 4685–4699. 10.1523/JNEUROSCI.0420-20.2020 32376782 PMC7294802

[B98] Silveira M. A. Drotos A. C. Pirrone T. M. Versalle T. S. Bock A. Roberts M. T. (2023). Neuropeptide Y signaling regulates recurrent excitation in the auditory midbrain. *J. Neurosci.* 43 7626–7641. 10.1523/JNEUROSCI.0900-23.2023 37704372 PMC10634549

[B99] Sokal R. R. Rohlf F. J. (2011). *Biometry.* New York, NY: W.H. Freeman.

[B100] Sperk G. Hamilton T. Colmers W. F. (2007). Neuropeptide Y in the dentate gyrus. *Prog. Brain Res.* 163 285–297. 10.1016/S0079-6123(07)63017-9 17765725

[B101] Suta D. Rybalko N. Pelánová J. Popeláø J. Syka J. (2011). Age-related changes in auditory temporal processing in the rat. *Exp. Gerontol.* 46 739–746. 10.1016/j.exger.2011.05.004 21609757

[B102] Syka J. (2020). “Age-related changes in the auditory brainstem and inferior colliculus,” in *Aging and hearing. springer handbook of auditory research*, eds Helfer K. S. Bartlett E. L. Popper A. N. Fay R. R. (Cham: Springer), 67–96.

[B103] Thompson A. M. (2005). “Descending connections of the auditory midbrain,” in *The inferior colliculus*, eds Winer J. A. Schreiner C. E. (New York, NY: Springer).

[B104] Wallace M. N. Shackleton T. M. Thompson Z. Palmer A. R. (2021). Juxtacellular labeling of stellate, disk and basket neurons in the central nucleus of the guinea pig inferior colliculus. *Front. Neural Circuits* 15:721015. 10.3389/fncir.2021.721015 34790099 PMC8592287

[B105] Walton J. P. (2010). Timing is everything: Temporal processing deficits in the aged auditory brainstem. *Hear. Res.* 264 63–69. 10.1016/j.heares.2010.03.002 20303402 PMC7045868

[B106] Walton J. P. Frisina R. D. O’Neill W. E. (1998). Age-related alteration in processing of temporal sound features in the auditory midbrain of the CBA mouse. *J. Neurosci.* 18 2764–2776. 10.1523/JNEUROSCI.18-07-02764.1998 9502833 PMC6793092

[B107] Walton J. P. Simon H. Frisina R. D. (2002). Age-related alterations in the neural coding of envelope periodicities. *J. Neurophysiol.* 88 565–578. 10.1152/jn.2002.88.2.565 12163510

[B108] Wenstrup J. J. (2005). “The tectothalamic system,” in *The inferior colliculus*, eds Winer J. A. Schreiner C. E. (New York, NY: Springer New York).

[B109] Wickham H. Averick M. Bryan J. Chang W. McGowan L. D. A. François R. (2019). Welcome to the tidyverse. *J. Open Source Softw.* 4:1686. 10.21105/joss.01686

[B110] Winer J. A. Larue D. T. Pollak G. D. (1995). GABA and glycine in the central auditory system of the mustache bat: Structural substrates for inhibitory neuronal organization. *J. Comp. Neurol.* 355 317–353. 10.1002/cne.903550302 7636017

[B111] Winer J. A. Saint Marie R. L. Larue D. T. Oliver D. L. (1996). GABAergic feedforward projections from the inferior colliculus to the medial geniculate body. *Proc. Natl. Acad. Sci. U. S. A.* 93 8005–8010. 10.1073/pnas.93.15.8005 8755593 PMC38865

[B112] Wood P. K Games P. (1990). Rationale, detection, and implications of interactions between independent variables and unmeasured variables in linear models. *Multivariate Behav. Res*. 25, 295–311. 10.1207/s15327906mbr2503_3 26761405

