# Supplementary material for: Neuropeptide Y mRNA expression in the aging inferior colliculus of fischer brown norway rats
Source: Front Aging Neurosci. 2025 Jul 23;17:1626021. doi: 10.3389/fnagi.2025.1626021 (PMC12326481; doi:10.3389/fnagi.2025.1626021)
Supplement: Supplementary file 1 [file Data_Sheet_1.pdf]

Table 1. Analysis of variance testing the influence of age and region on mRNA density[1].

Term	Statistic	*P*-value[2]
Overall Analysis of Variance		
Age	$F_{[3,17.2]} = 0.1011$	0.958
Region	$F_{[2,30.7]} = 19.34$	**<0.001*** **
Age-Region interaction	$F_{[6,30.6]} = 2.856$	**0.0250***
Pairwise comparisons – Age within Region		
Region = ICc		
3 months – 19 months	$t_{[17.4]} = -0.178$	0.9687
3 months – 24 months	$t_{[18.2]} = 0.040$	0.9687
3 months – 28 months	$t_{[17.4]} = 0.104$	0.9687
19 months – 24 months	$t_{[18.2]} = 0.222$	0.9687
19 months – 28 months	$t_{[17.4]} = 0.282$	0.9687
24 months – 28 months	$t_{[18.2]} = 0.067$	0.9687
Region = ICd		
3 months – 19 months	$t_{[17.4]} = 0.155$	0.9425
3 months – 24 months	$t_{[18.1]} = -0.073$	0.9425
3 months – 28 months	$t_{[17.3]} = 0.533$	0.9425
19 months – 24 months	$t_{[18.2]} = -0.232$	0.9425
19 months – 28 months	$t_{[17.4]} = 0.378$	0.9425
24 months – 28 months	$t_{[18.1]} = 0.621$	0.9425
Region = IClc		
3 months – 19 months	$t_{[17.9]} = 0.431$	0.8861
3 months – 24 months	$t_{[18.5]} = -0.583$	0.8861
3 months – 28 months	$t_{[17.7]} = 0.286$	0.8861
19 months – 24 months	$t_{[18.6]} = -1.026$	0.8861
19 months – 28 months	$t_{[17.8]} = -0.145$	0.8861
24 months – 28 months	$t_{[18.5]} = 0.878$	0.8861
Pairwise comparisons – Region within Age		
Age = 3 months		
ICc – ICd	$t_{[26.6]} = -3.841$	**0.0021****
ICc – IClc	$t_{[34.5]} = -1.066$	0.2938
ICd – IClc	$t_{[34.2]} = 2.538$	**0.0238****
Age = 19 months		
ICc – ICd	$t_{[29.7]} = -2.174$	0.0567
ICc – IClc	$t_{[37.7]} = 1.650$	0.1072
ICd – IClc	$t_{[38.5]} = 3.660$	**0.0023****
Age = 24 months		
ICc – ICd	$t_{[27.1]} = -4.358$	**0.0005*****
ICc – IClc	$t_{[37.4]} = -3.749$	**0.0009*****
ICd – IClc	$t_{[35.3]} = 0.274$	0.7854
Age = 28 months		
ICc – ICd	$t_{[25.8]} = -1.788$	0.2352
ICc – IClc	$t_{[32.0]} = -0.242$	0.8106

ICd – IClc	$t_{[31.4]} = 1.451$	0.2352

[1]Densities were square root transformed prior to analysis to improve normality.

[2]To mitigate the impact of heteroscedasticity among factor levels, degrees of freedom were adjusted using the Welch-Satterthwaite method. To control for multiple simultaneous comparisons, *p*-values for post-hoc pairwise comparisons were adjusted using the false discovery rate method (Benjamini and Hochberg, 1995). Significant tests following adjustment are indicated by bold typeface (* < 0.05; ** < 0.01; *** < 0.001).

Table 2. Analysis of variance testing the influence of age and region on NPY+/GAD+ density[1].

Term	Statistic	*P*-value[2]
Overall Analysis of Variance		
Age	$F_{[3,16]} = 0.2502$	0.86
Region	$F_{[2,38]} = 69.13$	**<0.001*** **
Pairwise comparisons – Age		
3 months – 19 months	$t_{[16]} = 0.756$	0.9804
3 months – 24 months	$t_{[16]} = 0.590$	0.9804
3 months – 28 months	$t_{[16]} = 0.731$	0.9804
19 months – 24 months	$t_{[16]} = -0.166$	0.9804
19 months – 28 months	$t_{[16]} = -0.025$	0.9804
24 months – 28 months	$t_{[16]} = 0.141$	0.9804
Pairwise comparisons – Region		
ICc – ICd	$t_{[38]} = -8.650$	**<0.001*** **
ICc – IClc	$t_{[38]} = 2.574$	**0.0141***
ICd – IClc	$t_{[38]} = 11.223$	**<0.001*** **

[1]Densities were rank transformed prior to analysis.

[2]To control for multiple simultaneous comparisons, *p*-values for post-hoc pairwise comparisons were adjusted using the false discovery rate method (Benjamini and Hochberg, 1995). Significant tests following adjustment are indicated by bold typeface (* < 0.05; ** < 0.01; *** < 0.001).

Table 3. Analysis of variance testing the influence of age and region on NPY+ density[1].

Term	Statistic	*P*-value[2]
Overall Analysis of Variance		
Age	$F_{[3,16]} = 2.727$	0.07851
Region	$F_{[2,38]} = 0.6077$	0.54980
Pairwise comparisons – Age		
3 months – 19 months	$t_{[16]} = -0.277$	0.7856
3 months – 24 months	$t_{[16]} = 0.413$	0.7856
3 months – 28 months	$t_{[16]} = 2.311$	0.1035
19 months – 24 months	$t_{[16]} = 0.690$	0.7503
19 months – 28 months	$t_{[16]} = 2.588$	0.1035
24 months – 28 months	$t_{[16]} = 1.898$	0.1518
Pairwise comparisons – Region		
ICc – ICd	$t_{[38]} = -0.562$	0.5921
ICc – IClc	$t_{[38]} = 0.540$	0.5921
ICd – IClc	$t_{[38]} = 1.102$	0.5921

[1]Densities were rank transformed prior to analysis.

[2]To control for multiple simultaneous comparisons, *p*-values for post-hoc pairwise comparisons were adjusted using the false discovery rate method (Benjamini and Hochberg, 1995). Significant tests following adjustment are indicated by bold typeface (* < 0.05; ** < 0.01; *** < 0.001).

Table 4. Analysis of variance testing the influence of age and region on GAD+ density[1].

Term	Statistic	*P*-value[2]
Overall Analysis of Variance		
Age	$F_{[3,16]} = 4.4609$	**0.01851***
Region	$F_{[2,38]} = 3.0016$	0.06161
Pairwise comparisons – Age		
3 months – 19 months	$t_{[16]} = 2.454$	0.0519
3 months – 24 months	$t_{[16]} = 3.408$	**0.0216***
3 months – 28 months	$t_{[16]} = 2.778$	**0.0403***
19 months – 24 months	$t_{[16]} = 0.954$	0.5317
19 months – 28 months	$t_{[16]} = 0.323$	0.7506
24 months – 28 months	$t_{[16]} = -0.630$	0.6450
Pairwise comparisons – Region		
ICc – ICd	$t_{[38]} = 1.473$	0.2235
ICc – IClc	$t_{[38]} = -0.959$	0.3435
ICd – IClc	$t_{[38]} = -2.432$	0.0595

[1]Densities were rank transformed prior to analysis.

[2]To control for multiple simultaneous comparisons, *p*-values for post-hoc pairwise comparisons were adjusted using the false discovery rate method (Benjamini and Hochberg, 1995). Significant tests following adjustment are indicated by bold typeface (* < 0.05; ** < 0.01; *** < 0.001).
